# Supplementary material for: HIVprotI: an integrated web based platform for prediction and design of HIV proteins inhibitors
Source: J Cheminform. 2018 Mar 9;10:12. doi: 10.1186/s13321-018-0266-y (PMC5845081; doi:10.1186/s13321-018-0266-y)
Supplement: Supplementary file 2 — Additional file 2. Source code of HIVProtI web server. [file 13321_2018_266_MOESM2_ESM.zip › HIVprotI_Source-code/hivprotI/weka-3-6-6/doc/weka/core/pmml/NormContinuous.html]

NormContinuous


---


|  |  |  |
| --- | --- | --- |
| **Overview** \| **Package** \| **Class** \| **Tree** \| **Deprecated** \| **Index** \| **Help** \| **Weka's home** | |  |
| **PREV CLASS**   **NEXT CLASS** | **FRAMES**    **NO FRAMES**     **All Classes** |  |
| SUMMARY: NESTED \| FIELD \| CONSTR \| METHOD | DETAIL: FIELD \| CONSTR \| METHOD |  |


---


## weka.core.pmml Class NormContinuous

```
java.lang.Object
  weka.core.pmml.Expression
      weka.core.pmml.NormContinuous
```

**All Implemented Interfaces:**: java.io.Serializable

---

``` public class NormContinuous extends Expression ```

Class encapsulating a NormContinuous Expression.

**Version:**
:   $Revision 1.0 $

**Author:**
:   Mark Hall (mhall{[at]}pentaho{[dot]}com)

**See Also:**: Serialized Form

---

**Constructor Summary**

`NormContinuous(org.w3c.dom.Element normCont,
FieldMetaInfo.Optype opType,
java.util.ArrayList<Attribute> fieldDefs)`


| **Method Summary** | |
| --- | --- |
| `double` | `getResult(double[] incoming)`             Get the result of evaluating the expression. |
| `java.lang.String` | `getResultCategorical(double[] incoming)`             Always throws an Exception since the result of NormContinuous must be continuous. |
| `double` | `getResultInverse(double[] incoming)`             Compute the inverse of the normalization (i.e. |
| `void` | `setFieldDefs(java.util.ArrayList<Attribute> fieldDefs)`             Set the field definitions for this Expression to use |
| `java.lang.String` | `toString(java.lang.String pad)` |

**Methods inherited from class weka.core.pmml.Expression**

`getExpression, getExpression, getFieldDef, getFieldDefIndex, getOptype, getResultContinuous, toString`

**Methods inherited from class java.lang.Object**

`equals, getClass, hashCode, notify, notifyAll, wait, wait, wait`

**Constructor Detail**

### NormContinuous

```
public NormContinuous(org.w3c.dom.Element normCont,
                      FieldMetaInfo.Optype opType,
                      java.util.ArrayList<Attribute> fieldDefs)
               throws java.lang.Exception
```

**Throws:**: `java.lang.Exception`


**Method Detail**

### setFieldDefs

```
public void setFieldDefs(java.util.ArrayList<Attribute> fieldDefs)
                  throws java.lang.Exception
```

:   Set the field definitions for this Expression to use

    :   **Overrides:**: `setFieldDefs` in class `Expression`
    :   **Parameters:**: `fieldDefs` - the field definitions to use **Throws:**: `java.lang.Exception` - if there is a problem setting the field definitions

---


### getResult

```
public double getResult(double[] incoming)
                 throws java.lang.Exception
```

:   Get the result of evaluating the expression. In the case
    of a continuous optype, a real number is returned; in
    the case of a categorical/ordinal optype, the index of the nominal
    value is returned as a double.

    :   **Specified by:**: `getResult` in class `Expression`
    :   **Parameters:**: `incoming` - the incoming parameter values **Returns:**: the result of normalizing the input field **Throws:**: `java.lang.Exception` - if there is a problem computing the result

---


### getResultInverse

```
public double getResultInverse(double[] incoming)
```

:   Compute the inverse of the normalization (i.e. map back to a unormalized value).

    :   **Parameters:**: `incoming` - the incoming parameter values **Returns:**: the unormalized value

---


### getResultCategorical

```
public java.lang.String getResultCategorical(double[] incoming)
                                      throws java.lang.Exception
```

:   Always throws an Exception since the result of NormContinuous must
    be continuous.

    :   **Specified by:**: `getResultCategorical` in class `Expression`
    :   **Parameters:**: `incoming` - the incoming parameter values **Returns:**: the result of evaluating the expression **Throws:**: `java.lang.Exception` - always

---


### toString

```
public java.lang.String toString(java.lang.String pad)
```

:   **Overrides:**: `toString` in class `Expression`


---


|  |  |  |
| --- | --- | --- |
| **Overview** \| **Package** \| **Class** \| **Tree** \| **Deprecated** \| **Index** \| **Help** \| **Weka's home** | |  |
| **PREV CLASS**   **NEXT CLASS** | **FRAMES**    **NO FRAMES**     **All Classes** |  |
| SUMMARY: NESTED \| FIELD \| CONSTR \| METHOD | DETAIL: FIELD \| CONSTR \| METHOD |  |


---
